# Effects of Dynamin-related Protein 1 Regulated Mitochondrial Dynamic Changes on Invasion and Metastasis of Lung Cancer Cells

**DOI:** 10.7150/jca.29756

**Published:** 2019-07-08

**Authors:** Jie-Tao Ma, Xiang-Yan Zhang, Rui Cao, Li Sun, Wei Jing, Jian-Zhu Zhao, Shu-Ling Zhang, Le-Tian Huang, Cheng-Bo Han

**Affiliations:** Department of Oncology, Shengjing Hospital of China Medical University, Shenyang 110022, China

**Keywords:** dynamin-related protein 1, invasion, lung adenocarcinoma, metastasis, mitochondrial fission

## Abstract

**Objective**: Mitochondrial imbalance of division and fusion will lead to uncontrolled cell growth. This study investigated the effects of mitochondrial dynamics regulated by dynamin-related protein 1 (Drp1) on the invasion and metastasis of lung cancer cells at the cellular level.

**Methods**: Lentivirus-mediated RNAi and gene overexpression vectors containing shDrp1 and Lv-Drp1 were transfected into lung adenocarcinoma cell lines 95D and A549, respectively. An MTT assay was used to assess cell viability and a cell clone assay was used to evaluate the tumorigenic ability of lentivirus-infected cells. Cell invasion and wound healing assays were used to assess cell invasiveness and the migration rate after lentivirus infection. Annexin V-APC staining was used to determine the cell apoptosis rate.

**Results**: In 95D cells, when the Drp1 gene is overexpressed (OE) the proliferation rate and apoptosis rate were significantly higher than those in the control group (NC_OE_) (P < 0. 05). There was no significant difference in clone number, invasion rate, and migration rate between the two groups (P > 0. 05). The proliferation rate and clone number in the shDrp1 infected 95D cell group (KD) were significantly lower than those in the control group (NC_KD_) (P < 0. 05). There was no difference in apoptosis rate, invasion rate, and migration rate between h (P > 0.05). In A549 cells, unlike in 95D cells, the invasion rate of the KD group was 25% lower than that of the NC_KD_ group (P < 0.05). After 8 hours, the cell migration rates of the two groups were basically the same, but after 24 hours, the migration rate of the KD group was 10% lower than that of the NC_KD_ group (P < 0.05). Compared with the NC_OE_ group, the migration rate of the OE group increased significantly (P < 0.05).

**Conclusion**: Mitochondrial Drp1 is associated with the proliferation, invasion, and metastasis of lung adenocarcinoma cells. Inhibition of Drp1 expression may contribute to anti-tumor therapy for lung cancer.

## Introduction

Lung cancer is the most common malignant tumor and the leading cause of cancer-related deaths in the world 1. About two-thirds of patients with lung cancer have lost the opportunity for surgery by the time of the initial diagnosis. Metastasis is the most important biological behavior of cancer and the main cause of death. The mechanism of metastasis involves the adhesion, migration, and movement of cancer cells and the degradation of the extracellular matrix 2. The metastasis of cancer is quite complex, and many problems remain unclear.

In mammals, mitochondria are the only organelles with genetic material outside the nucleus. Early studies have shown that mitochondria are not only the energy factory of cells but also play a key role in the process of cell apoptosis 3,4. Many studies, including our previous studies, have shown that mitochondrial DNA (mtDNA) variations (mutations, instability, and increased copy number) play an important role in tumorigenesis, progression, and differentiation 5-8.

Studies have shown that mitochondria are highly dynamic organelles that respond to the physiological needs of cells 9,10. Through continuous movement, division, and fusion, mitochondria form mitochondrial reticulum/networks in cells. Dynamin-related protein 1 (Drp1) and optic atrophy protein 1 (Opa1) are involved in the regulation of mitochondrial morphological changes in two opposite processes: division and fusion 11,12. Unbalanced fission and fusion lead to uncontrolled cell growth regulation, which may be related to tumorigenesis and progression 13-15. Our previous study found that the dynamic distribution of mitochondria in cancer cells may be related to invasion and migration 16. In that study, we observed that the chemokine CXCL12 not only enhanced the invasion and migration of A549 lung adenocarcinoma cells but also significantly changed the distribution of mitochondria and caused a significant polarization change in the distribution of mitochondria. However, CXCL12 did not enhance the invasiveness and migration of ρ^0^A549 cells (a constructed A549 cell line lacking mtDNA), and the distribution of mitochondria in the cells did not change significantly.

Basic studies have shown that the expression of the Drp1 protein in A549 lung adenocarcinoma cells is higher than that in normal airway cells. Drp1 inhibitor mitochondrial division inhibitor (mdivi-1) can inhibit the proliferation of cancer cells 17. The expression levels of total Drp1 and phosphorylated Drp1 (phosphorylation at Ser-616 enhances Drp1 activity) in lung adenocarcinoma tissue were significantly higher than those in adjacent normal lung tissue 17. Nevertheless, it is unclear whether the mitochondrial morphological changes regulated by Drp1 are involved in the invasion and metastasis of tumor cells. The aim of this study was to investigate the effects of mitochondrial morphological changes regulated by mitochondrial Drp1 on the proliferation, invasion, and metastasis of tumor cells in vitro.

## Materials and methods

### Reagents

Methyl thiazolyl tetrazolium (MTT) (Catalog (cat.) No. JT343, Genview, CA USA), Annexin V Apoptosis Detection Kit APC (cat.No.88-8007, Thermo Fisher Scientific Inc. Carlsbad, CA USA), Corning BioCoat Matrigel Invasion Chambers (cat.No.354480, Corning, NY USA), and a CellTiter 96 AQueous One Solution Cell Proliferation Assay (MTS) Kit (cat. No. G3580, Promega Corporation, Madison, WI USA) were purchased from the indicated sources.

### Vector construction

**Figure 1:**
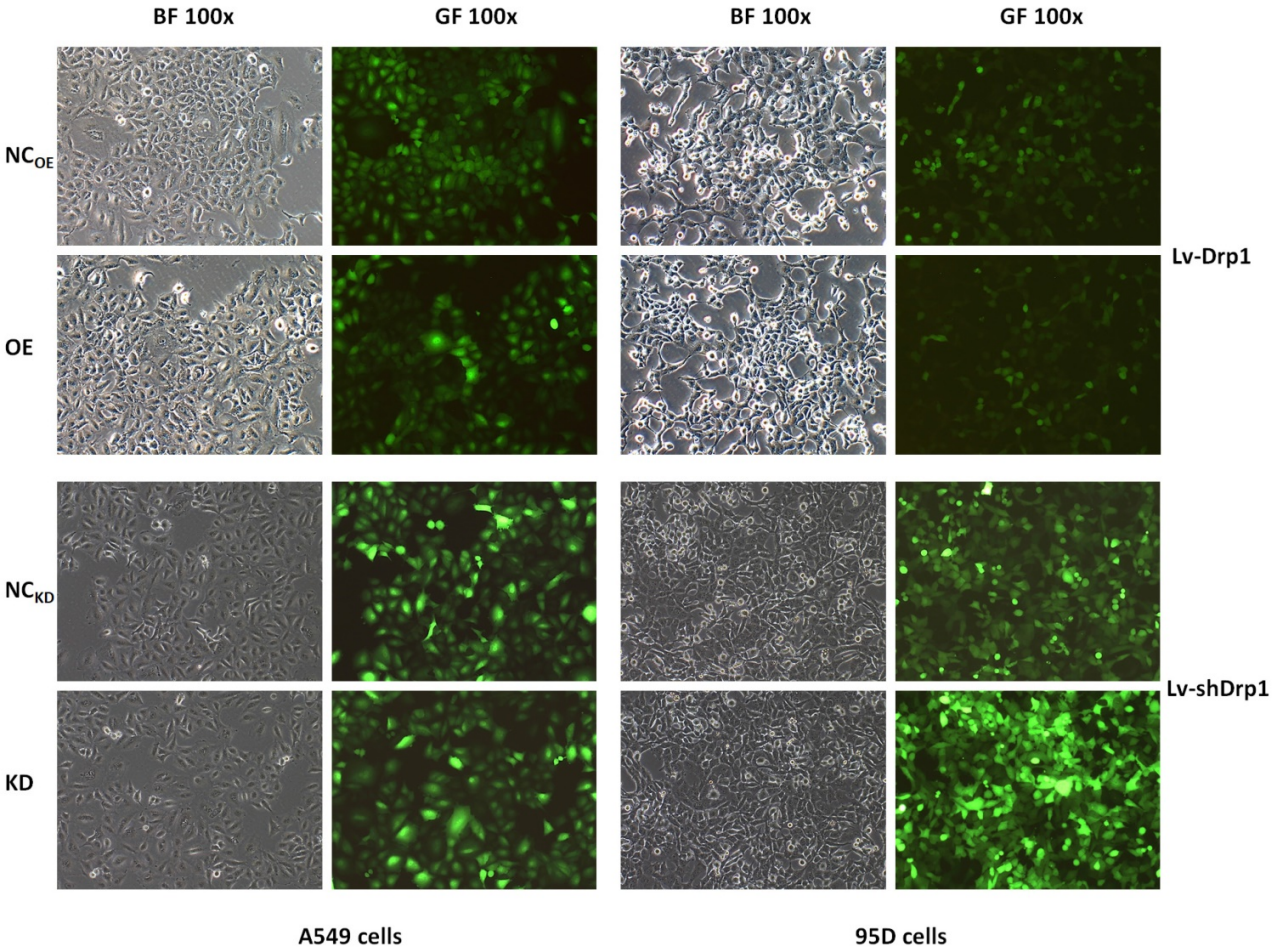
Lung cancer cells were transfected with Lv-Drp1 and Lv-shDrp1. The overexpression or gene knockdown of Drp1 in 95D and A549 lung cancer cell lines were observed by bright visual fields (BF) and green fluorescent visual fields (GF). NC: normal target cells, cells infected with negative control virus; OE: normal target cells, cells infected with the virus of target gene overexpression. KD: normal target cells, cells infected with shRNA virus of the Drp1 gene.

The Drp1 overexpressing lentiviral vector (Lv-Drp1) and Drp1 knockdown lentiviral vector (Lv-shDrp1) were constructed by Shanghai Genechem Co., Ltd. The Drp1 gene was the human DNM1L (Dynamin-1-like gene), GENE_ID 10059, GenBank NM_012063. The lentiviral negative control (NC) were NC_OE_ (CON238) and NC_KD_ (CON053) (Shanghai Genechem), respectively. The constructed vectors (Lv-Drp1 and Lv-shDrp1) were confirmed by qPCR, sequencing, and Western blotting. The expression and interference efficiency of the target genes were verified by RT-PCR, and the stable expression strain was screened for subsequent functional experiments (Fig. 1). The research subgroups were as follows: a Drp1 overexpression (OE, Lv-Drp1) group; a Drp1 knockdown (KD, Lv-shDrp1) group; and a negative control (NC) group (normal target cells, plus NC lentiviral viral-infected cell groups).

### Cell lines and cell cultures

The 95D human lung cancer high metastatic cell line and A549 lung adenocarcinoma cell lines were purchased from Shanghai Institute of Cell Research (Cell Resource Center, Shanghai Institutes for Biological Sciences, Chinese Academy of Sciences). These cells were cultured at 37℃in a humidified atmosphere containing 5% CO_2_ in Dulbecco's modified Eagle's medium (cat.No.10-013-CVR, Corning Inc., USA) supplemented with 10% fetal bovine serum (FBS, Life Technologies).

### Cell viability assay

Cell viability was assessed using the MTT assay to verify the effects of the target gene on cell proliferation. Each group of cells in the logarithmic growth phase was trypsinized, and then resuspended in a complete medium to form a cell suspension, and seeded into a 96-well plate at 2.5 × 10^3^ cells per well, then incubated with 20 μL of 5 mg/mL MTT for 4 h. The culture solution was completely aspirated and 100 μL of dimethyl sulfoxide (Shanghai Trial Chemical Reagent Co., Ltd.) was added. OD value at 490 nm was measured.

### Clonogenic assay

A cell cloning assay was used to evaluate the tumorigenic ability of lentivirus-infected cells. Cells were seeded in 6-well culture plates at 5.0 × 10^2^ cells per well, and cultured for 14 days. There were three replicate wells in each group. After fixation by paraformaldehyde for 30-60 min, cells were stained with Giemsa dye solution for 10-20 min. Cells were washed several times with ddH_2_O and then dried, photographed, and counted to obtain the number of clones. A colony consisted of at least 50 cells.

### Apoptosis assay

Apoptosis was detected by an APC Annexin V Apoptosis Detection Kit according to the manufacturer's instructions. In brief, cells in the exponential growth phase were digested with trypsin, resuspended in complete medium, centrifuged at 1300 rpm and the supernatant was then discarded. The cell pellet was washed with 4 °C pre-cooled D-Hanks solution (Shanghai Genechem Co., Ltd.), and once with 1× binding buffer, then centrifuged at 1300 rpm for 3 min. The cell pellet was resuspended in 200 μL of 1× binding buffer, stained with 10 μL of Annexin V-APC, and protected from light for 10-15 min at room temperature.

### Invasion assay

The invasion assay was performed using Corning BioCoat Matrigel Invasion Chambers according to the manufacturer's instructions. In brief, invasion chambers were removed at -20 °C, placed on a 24-well plate on a sterile operating table and returned to room temperature. Serum-free medium (500 μL) was added to the upper and lower chambers then placed in a 37 °C incubator for 2 h to rehydrate the Matrigel matrix. A serum-free cell suspension containing 10^5^ cells per well was prepared. Cells were stained with Giemsa solution, and invading cells were counted by photographing the membrane under a microscope.

### Cell wound healing assay

The migration ability of tumor cells after lentivirus infection was observed using a cell wound healing assay. Infected cells (3 × 10^4^) were added to the wells, and the low concentration serum medium was replaced the next day. The central part of the 96-well plate was aligned with a scratch tester and gently pushed upward to form a scratch. The cells were gently rinsed 2-3 times with serum-free medium and cultured in a 5% CO_2_ incubator at 37 °C. Images were taken under a microscope at 0, 8, and 24 h after migration. The width of the wound at different time points was measured to compare the migration ability of tumor cells.

### Statistical analysis

All data are presented as the mean ± standard deviation (SD). Statistical analyses were performed using the SPSS statistical software program, version 19.0 (SPSS, Chicago, IL, USA). P values were obtained using a two-tailed t-test, and P < 0.05 was considered statistically significant.

## Results

### Cell proliferation

**Figure 2:**
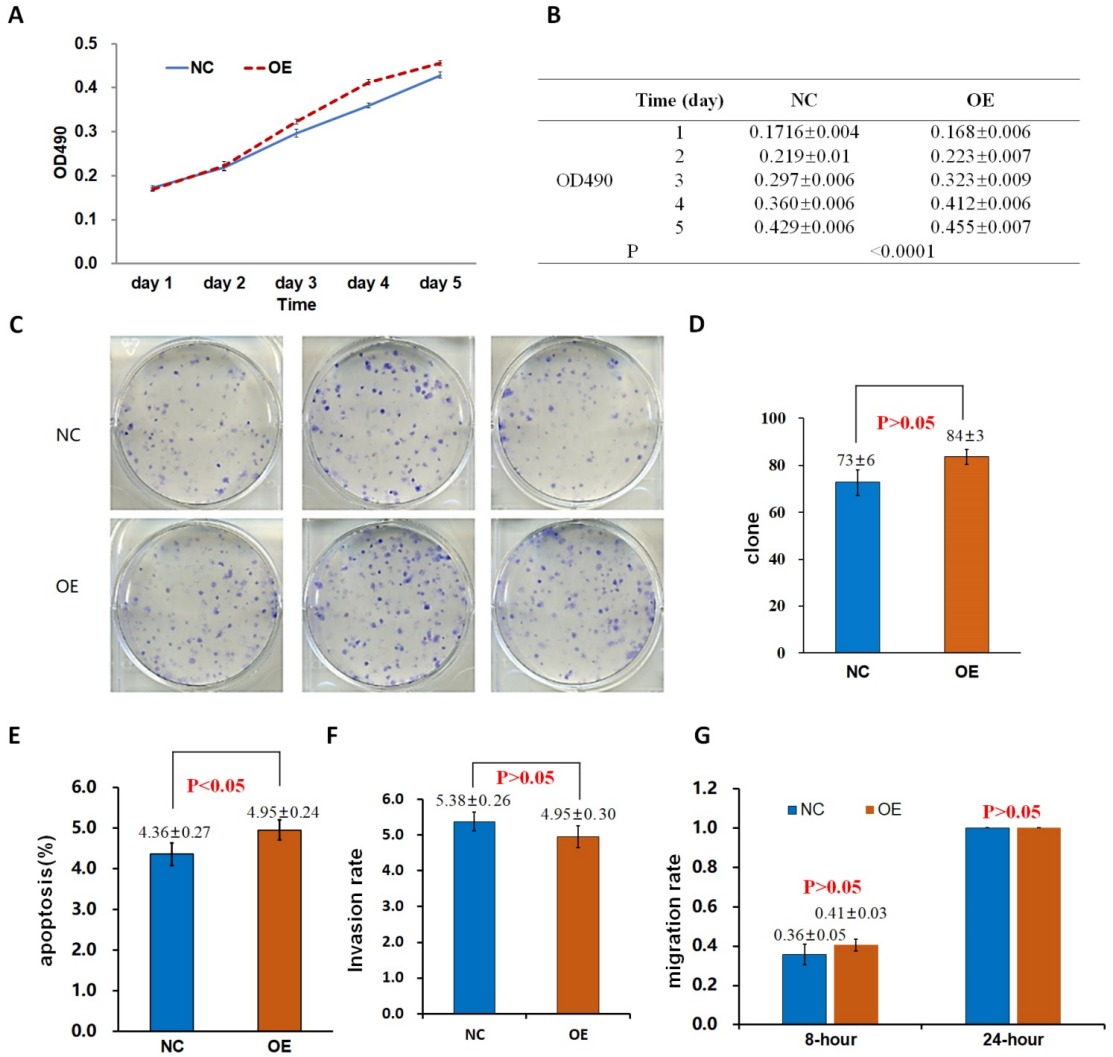
Effects of the overexpression of Drp1 (Lv-Drp1) on cell viability (A, B), tumorigenic capacity (C, D), apoptosis (E), invasion (F), and migration (G) of 95-D lung cancer cells. NC: normal target cells, cells infected with negative control virus; OE: normal target cells, cells infected with the virus of target gene overexpression.

**Figure 3:**
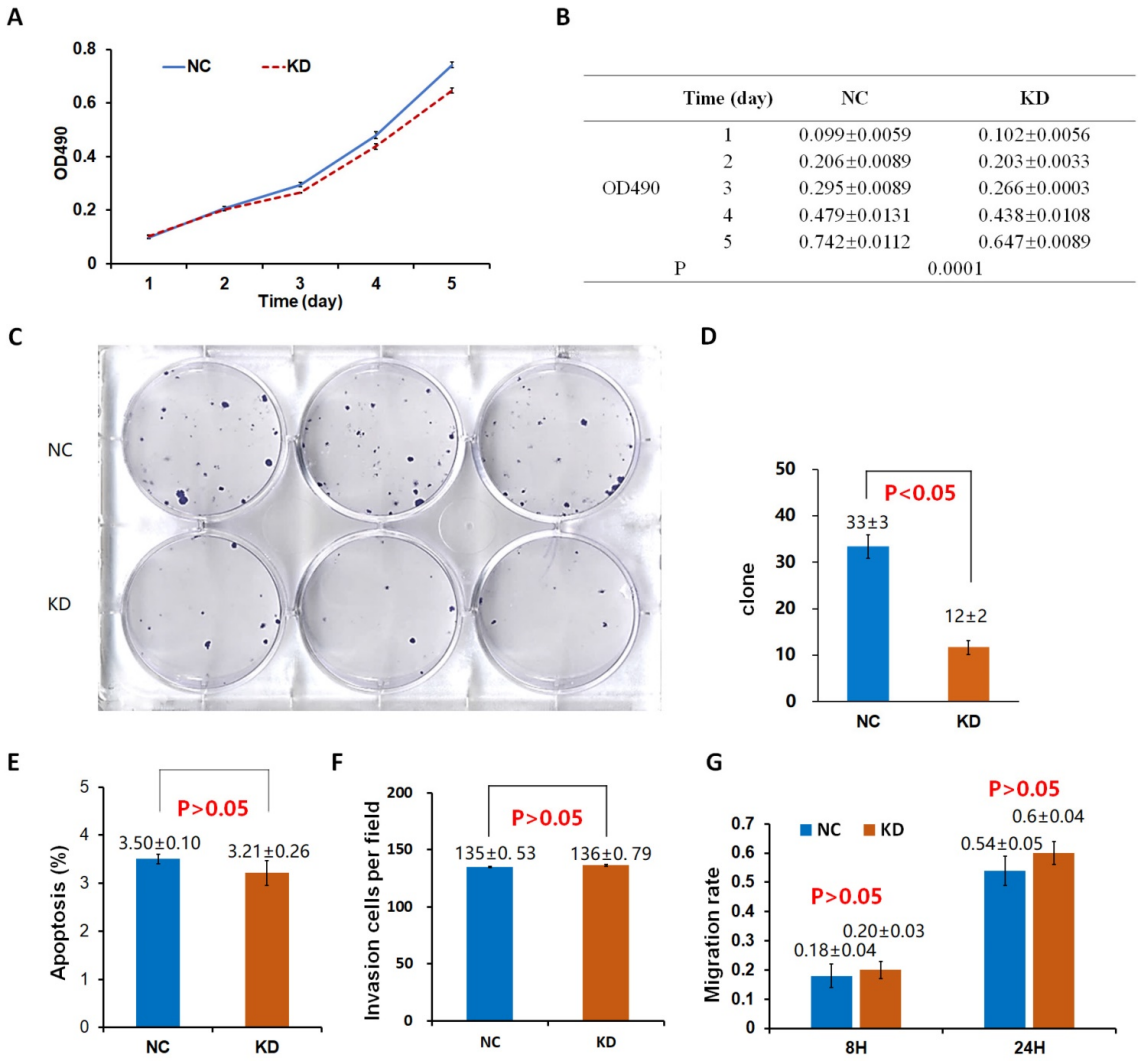
Effects of Drp1 knockdown (Lv-shDrp1) on cell viability (A, B), tumorigenic capacity (C, D), apoptosis (E), invasion (F), and migration (G) of 95D lung cancer cells. NC: normal target cells, cells infected with negative control virus; KD: normal target cells, cells infected with shRNA virus of the Drp1 gene.

**Figure 4:**
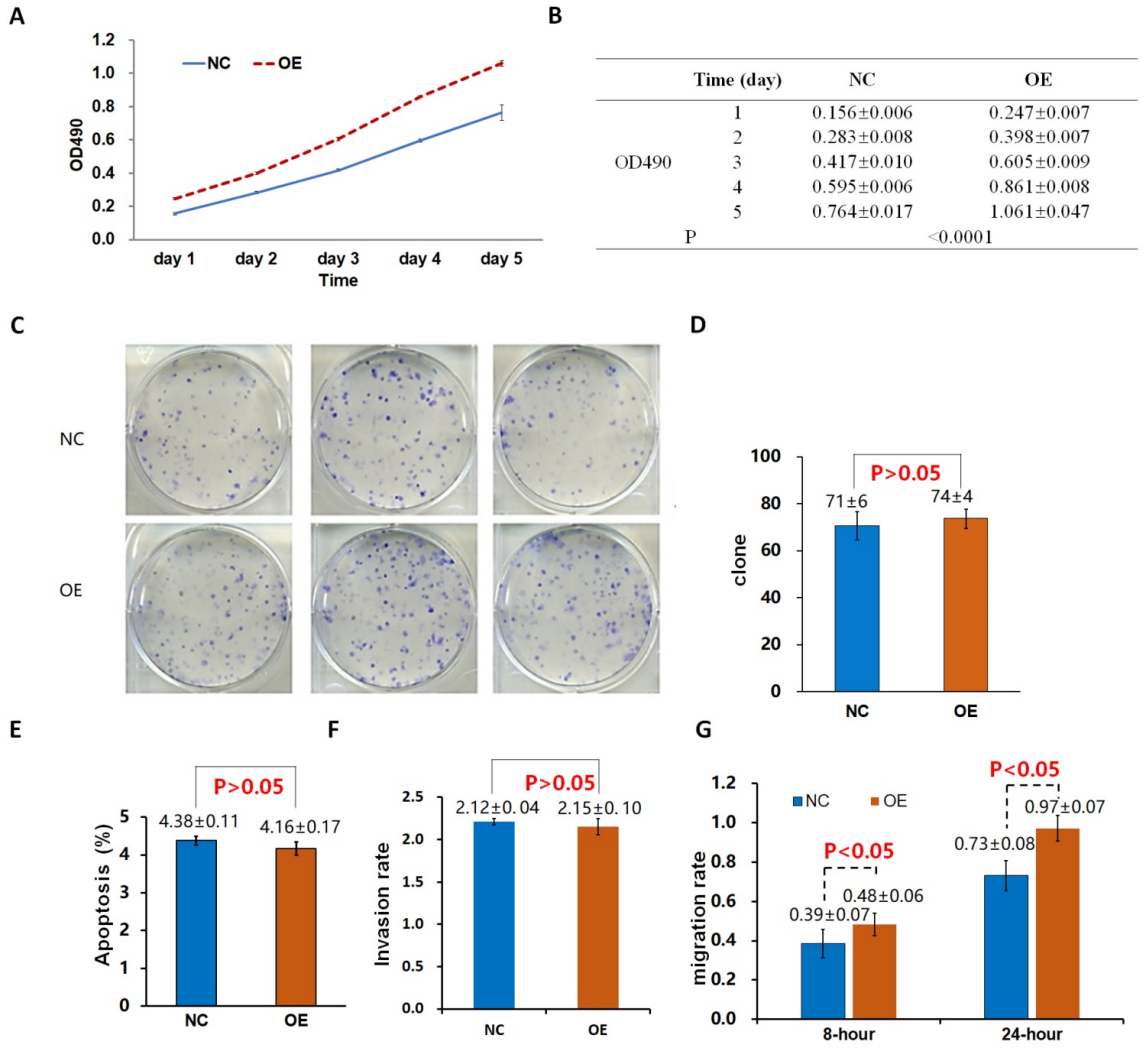
Effects of Lv-Drp1 on cell viability (A, B), tumorigenic capacity (C, D), apoptosis (E), invasion (F), and migration (G) of A549 lung cancer cells.

**Figure 5:**
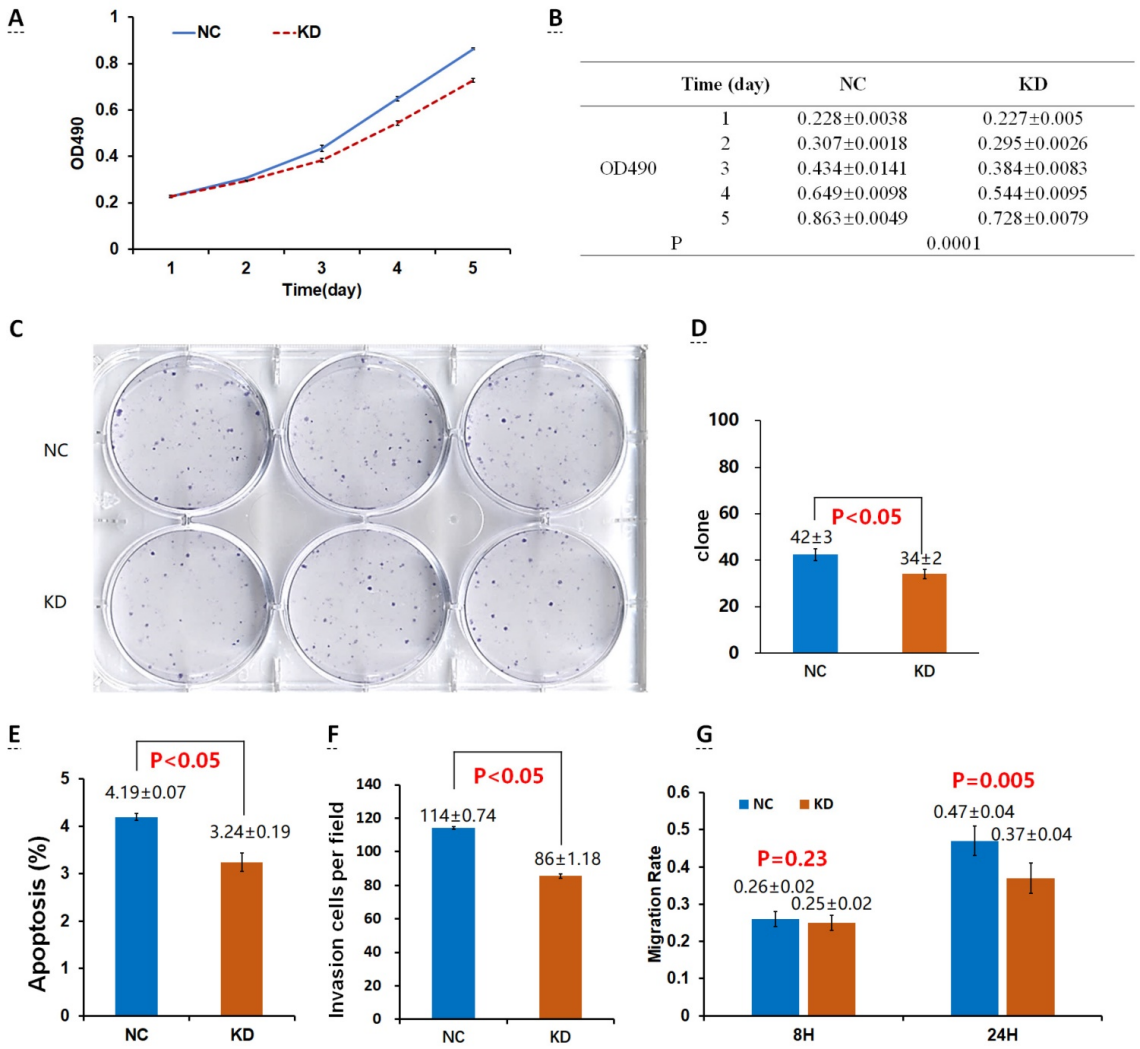
Effects of Lv-shDrp1 on cell viability (A, B), tumorigenic capacity (C, D), apoptosis (E), invasion (F), and migration (G) of A549 lung cancer cells.

The proliferation rate of 95D cells in the OE group was significantly higher than that in the control group (NC_OE_) on the 4th and 5th day (P < 0.05; Fig. 2A, B). After the shDrp1 lentivirus-infected 95D lung cancer cell line was cultured for 5 days, the cell proliferation rate of the KD group was significantly lower than that of the control group (NC_KD_; P < 0.05; Fig. 3A, B). Similar results were obtained for A549 cells (Fig. 4A, B; Fig. 5A, B).

### Cell clonogenic ability

There was no significant difference in the number of clones between the OE group and the NC_OE_ group (P > 0.05) in Lv-Drp1 infected 95D cells or A549 cells (Fig. 2C, D; Fig. 4C, D). For Lv-shDrp1 infected 95D and A549 lung cancer cells, the number of clones in the KD group was significantly lower than that in the NC_KD_ group (P < 0.05; Fig. 3C, D; Fig. 5C, D).

### Cell apoptosis

For 95D lung cancer cells infected with lv-drp1, the apoptosis rate in the OE group was slightly higher than that in the NC_OE_ group (P<0.05; Fig. 2E). For A549 cells, there was no significant change in the apoptosis rate between the OE group and the NC_OE_ group (P > 0.05; Fig. 4E). For 95D cells infected with Lv-shDrp1, the apoptosis rate was lower than 5% in both the KD group and the NC_KD_ group, and no significant apoptosis was observed (P > 0.05; Fig. 3E). For A549 cells, the apoptosis rate in the KD group was lower than that in NC_KD_ group (P < 0.05), but the apoptosis rate in both groups was less than 5% (Fig. 5E).

### Cell invasive ability

There was no significant difference in the invasive rate between the OE group and the NC_OE_ group for Lv-Drp1 infected 95D cells or A549 cells, P>0.05 (Fig. 2F, Fig. 4F). After 95D cells were infected by Lv-shDrp1 for 24 h, the invasion rate of 95D cells in the KD group and NC_KD_ group was basically the same, with no decrease (Fig. 3F). However, after A549 cells were infected with Lv-shDrp1 for 24 h, the invasion rate of the KD group was 25% lower than that of the NC_KD_ group (P < 0.05; Fig. 5F).

### Cell migration ability

For 95D cells infected with Lv-Drp1, there was no significant difference in cell migration rate between the OE group and the NC_OE_ group after 8 h (P> 0.05). However, after 24 h, in both the OE and NC_OE_ groups the wound had healed and the difference in cell mobility could not be distinguished (Fig. 2G, Fig. S1). For A549 cells, the cell migration rate in the OE group was significantly higher than that in the NC_OE_ group (P<0.05; Fig. 4G, Fig. S2). For 95D cells infected by Lv-shDrp1, the cell migration rates of the KD group and the NC_KD_ group were basically the same after 8 and 24 h (P > 0.05; Fig. 3G, Fig. S3). For A549 cells, the cell migration rates of the two groups were basically the same after 8 h, but after 24 h, the cell migration rate of the KD group decreased by 10% compared to the NC_KD_ group (P<0.05; Fig. 5G, Fig. S4).

## Discussion

Mitochondrial morphological changes are precisely regulated by multiple proteins 18. Drp1 is the most important highly conserved guanosine triphosphatase (GTPase) involved in the regulation of mitochondrial fission. The upregulation of Drp1 activity leads to mitochondrial fission, whereas inhibition or downregulation of Drp1 can trigger the formation of a highly intertwined mitochondrial network 19. In the present study, siRNA-mediated Drp1 knockdown significantly reduced cell proliferation and colony formation in both 95D and A549 cells. In addition, following Drp1 knockdown, the invasion rate and migration rate of A549 cells after 24 h decreased by 25% and 10%, respectively, compared with the control group, whereas the overexpression of Drp1 significantly increased the cell migration rate. These results suggest that the mitochondrial fission protein Drp1 is associated with the proliferation, invasion, and metastasis of lung adenocarcinoma.

The morphology of mitochondria is an important factor in determining mitochondrial function. Excessive fission and fusion results in the changes of mitochondrial fragmentation and elongation, respectively 17-19. Mitochondrial fission mediated by Drp1 upregulation is related to the cellular transformation of oncogene RAS. RAS-driven mitochondrial over-fragmentation is critical for cancer metabolism and cell transformation, and knocking down or inhibiting Drp1 can abolish or terminate the transformation 20. The excessive fission of mitochondria has been found to be associated with carcinogenesis, apoptosis, and proliferation, and even drug resistance 21, in many cancers, such as colorectal cancer 21,23, thyroid cancer 24, and cutaneous squamous cell carcinoma 25. Our study found that Drp1 knockdown significantly reduced the proliferation and clonal formation of lung adenocarcinoma cells, which is consistent with the findings of Rehman et al. They found that the inhibition of Drp1 expression reduced N-cell xenografts in nude mice and inhibited tumor proliferation 17.

Mitochondrial division plays an important role in the release of apoptosis-promoting factors (proapoptotic factors) into the intermembrane cavities and the intracellular transport of mitochondria mediated by the cytoskeleton. The fused mitochondrial network can dissipate the energy generated by metabolism through the transmission of membrane potential 26. Inoue-Yamauchi et al. 23 found that siRNA-mediated Drp1 knockdown could promote elongated mitochondria accumulation in human colon cancer cells (HCT116 and SW480 cells) in vitro, and down-regulated Drp1 resulted in an increased cytochrome C (cyt-c) release and a greater than 30% increase in apoptosis rate. The results showed that Drp1 could inhibit the apoptosis of colon cancer cells by regulating the release of cyt-c and the integrity of the mitochondrial membrane. Our study showed that the overexpression or knockdown of Drp1 did not independently induce apoptosis in lung cancer cells because all apoptosis rates were less than 5%. Although the study by Rehman et al. showed that Drp1 knockdown increased the apoptosis rate of lung cancer A549 cells by 3-4 times, the apoptosis rates were all less than 3% 17. Studies have also shown that Drp1 deletion inhibits mitochondrial fragmentation and cyt-c release induced by apoptotic factors 27,28. Therefore, how the Drp1 protein participates in the apoptosis of cancer cells needs to be confirmed by further intervention experiments.

The intracellular distribution of mitochondria is a process of movement along microtubules, which is associated with the mitochondrial morphological changes regulated by mitochondrial fission and fusion proteins Drp1 and Opa1 29. The mitochondrial network must be split into smaller organelles for transport, and this transport is regulated by motor proteins (or molecular motors) with directional characteristics. Motor proteins, including kinesin and dynein, can convert the chemical energy of ATP high-energy phosphate bonds into mechanical energy, enabling cells to move 30,31. The mitochondrial network distributed as an electrokinetic complex can transmit the mitochondrial membrane potential produced by proton pumps in the electron respiratory chain. This mechanism is particularly important in dissipating ATP energy generated by cell metabolism 30,32.

In a study of T cell chemotaxis, Campello et al. 33 found that the chemokine CXCL12 could make the mitochondria change the polarity of tail distribution and induce the cell migration of T cells transfected with Drp1. It was suggested that the redistribution (polarization changes) of mitochondria in cells can drive the chemotaxis and migration of lymphocytes. Our previous studies on lung cancer cell lines have shown that mitochondrial polarity changes may be related to the invasion and migration of cancer cells 16. In this study, we found that the invasion rate and migration rate (after 24 h) of lung cancer A549 cells following Drp1 knockdown decreased by 25% and 10%, respectively, when compared with the control group, whereas overexpression of Drp1 significantly increased the cell migration rate, indicating that Drp1 is associated with the invasion, migration, and metastasis of lung cancer cells. The mechanism of this process may be that mitochondrial morphological changes regulated by Drp1 and the effect of the microtubule molecular motor, causes polarity changes and the redistribution of mitochondria in lung cancer cells, thus participating in the invasion and metastasis of lung cancer.

There are some limitations to this study. Only the 95D human lung cancer high metastatic cell line and A549 lung adenocarcinoma cell lines were used for in vitro assays. The lack of normal cells, other tumor cells, and xenograft models possibly makes it insufficient to judge the specificity of the in vitro findings. In addition, the detailed mechanisms of the inhibitory effect of Drp1 knockdown on lung cancer cells were not evaluated in this study and need further research in future study.

## Conclusion

Our study demonstrates that Drp1 is involved in the regulation of proliferation, invasion, and migration of lung cancer cells in vitro, and inhibiting the signal pathway of Drp1 will contribute to the development of an anti-tumor therapy for lung cancer. However, the underlying mechanisms and clinical applications need further study, and relevant studies are being conducted on the regulatory signal pathway of Drp1.

## Supplementary Material

Supplementary figures and tables.

## Abbreviations

mtDNA: mitochondrial DNA
CXCL12: chemokine (C-X-C motif) ligand 12
MTT: methyl thiazolyl tetrazolium
Drp1: dynamin-related protein 1
Opa1: optic atrophy protein 1
Lv-Drp1: Drp1 overexpressing lentiviral vector
Lv-shDrp1: Drp1 knockdown lentiviral vector
DNM1L: Dynamin-1-like gene
NC: negative control
OE: overexpression
KD: knockdown
SD: standard deviation
GTPase: guanosine triphosphatase
