# Supplementary material for: Effects of Dynamin-related Protein 1 Regulated Mitochondrial Dynamic Changes on Invasion and Metastasis of Lung Cancer Cells
Source: J Cancer. 2019 Jul 8;10(17):4045–53. doi: 10.7150/jca.29756 (PMC6692611; doi:10.7150/jca.29756)
Supplement: Supplementary file 1 — Supplementary figures and tables. [file jcav10p4045s1.pptx]

## Slide 1
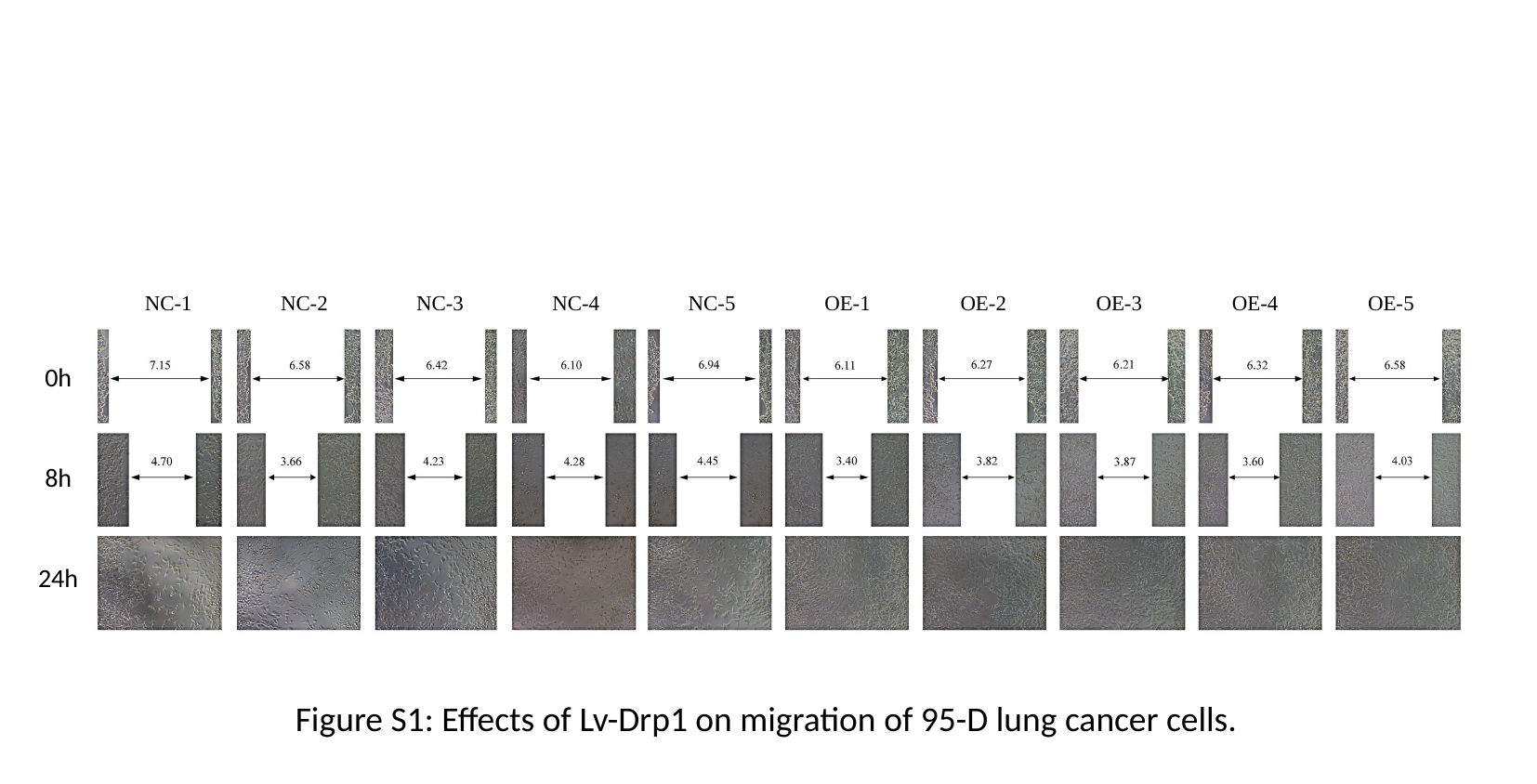

| NC-1 | NC-2 | NC-3 | NC-4 | NC-5 | OE-1 | OE-2 | OE-3 | OE-4 | OE-5 |
| --- | --- | --- | --- | --- | --- | --- | --- | --- | --- |
| 0h |
| --- |
| 8h |
| 24h |
Figure S1: Effects of Lv-Drp1 on migration of 95-D lung cancer cells.

## Slide 2
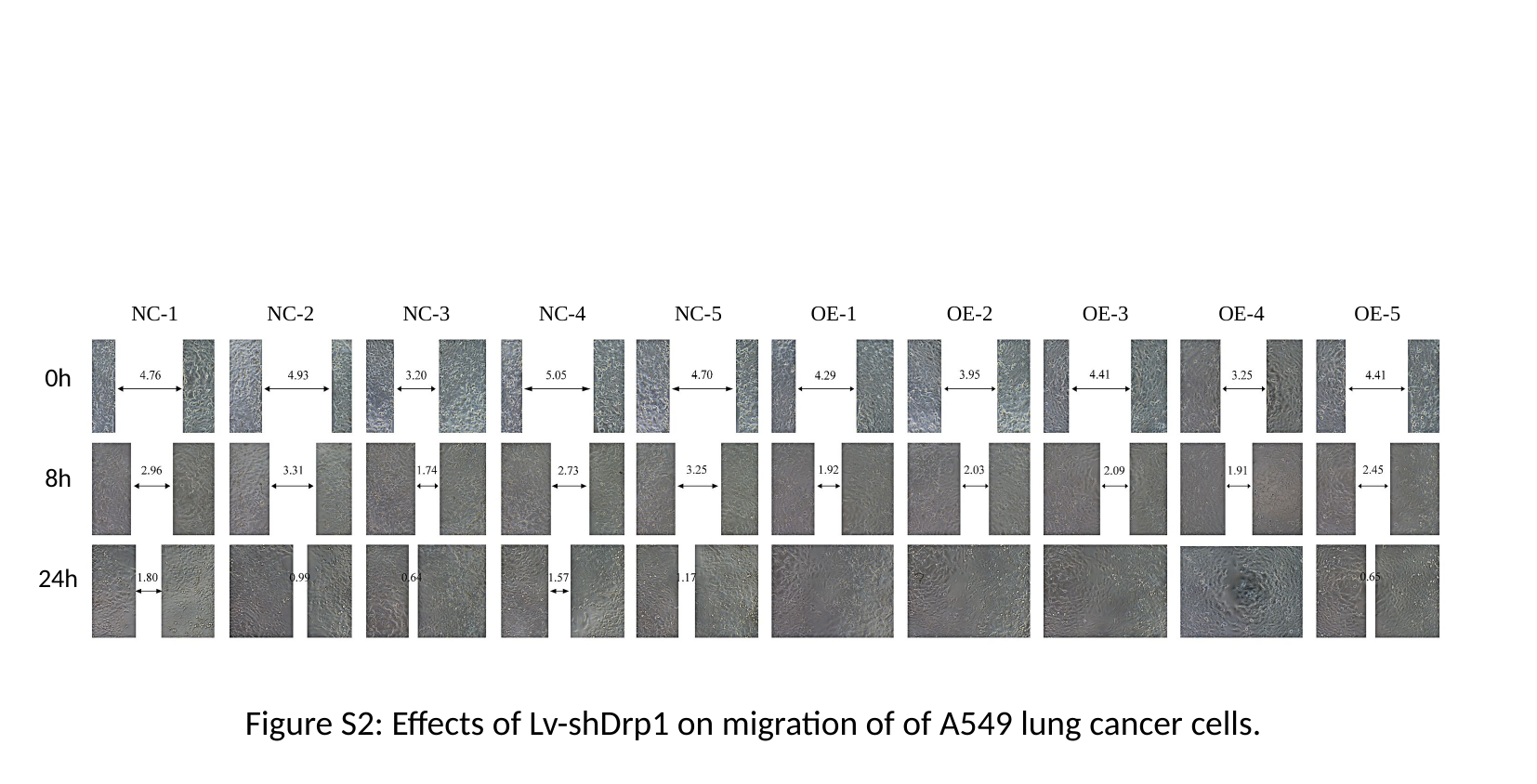

| NC-1 | NC-2 | NC-3 | NC-4 | NC-5 | OE-1 | OE-2 | OE-3 | OE-4 | OE-5 |
| --- | --- | --- | --- | --- | --- | --- | --- | --- | --- |
| 0h |
| --- |
| 8h |
| 24h |
Figure S2: Effects of Lv-shDrp1 on migration of of A549 lung cancer cells.

## Slide 3
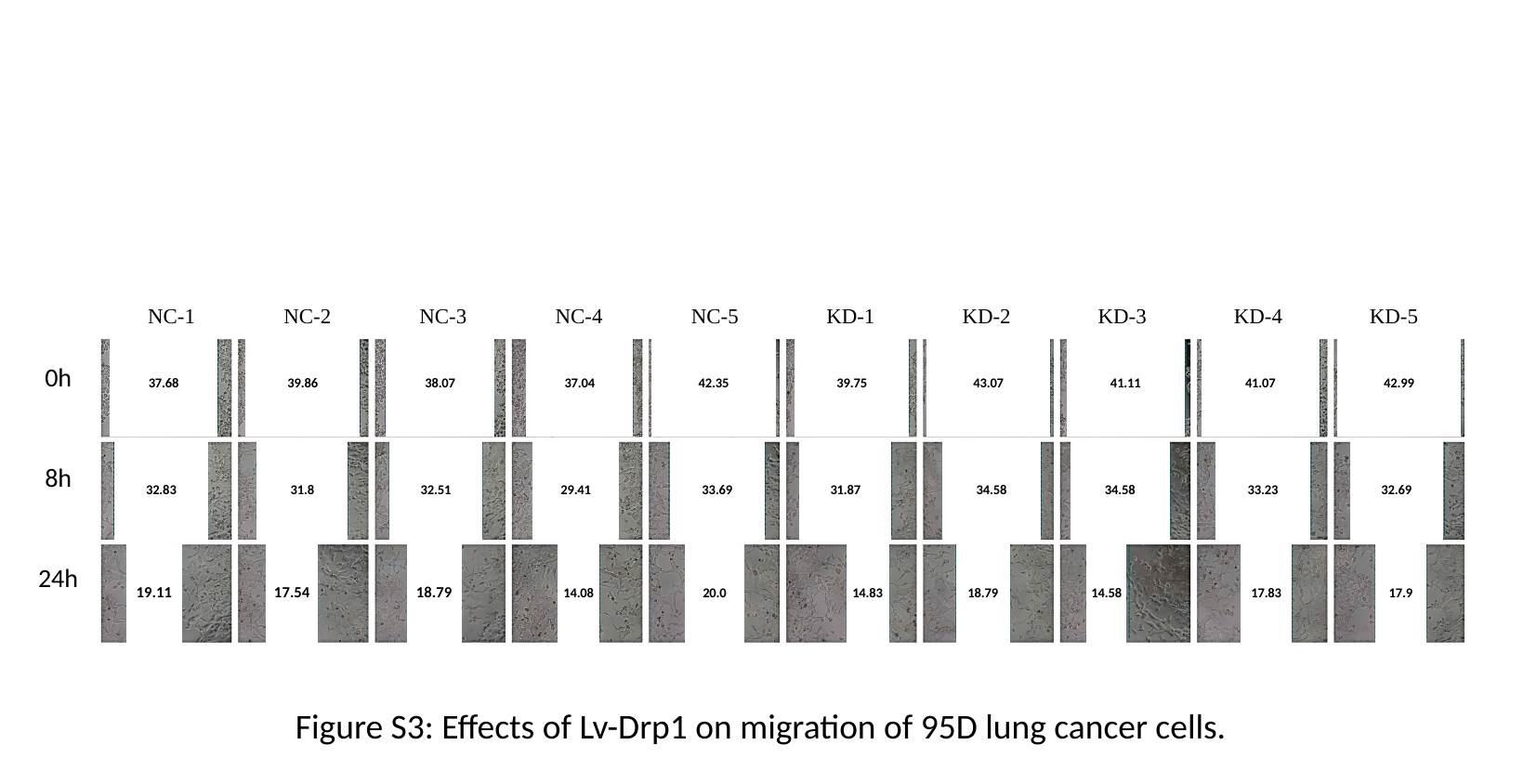

| NC-1 | NC-2 | NC-3 | NC-4 | NC-5 | KD-1 | KD-2 | KD-3 | KD-4 | KD-5 |
| --- | --- | --- | --- | --- | --- | --- | --- | --- | --- |
| 0h |
| --- |
| 8h |
| 24h |
| 37.68 |
| --- |
| 39.86 |
| --- |
| 38.07 |
| --- |
| 37.04 |
| --- |
| 42.35 |
| --- |
| 39.75 |
| --- |
| 43.07 |
| --- |
| 41.11 |
| --- |
| 41.07 |
| --- |
| 42.99 |
| --- |
| 32.83 |
| --- |
| 31.8 |
| --- |
| 32.51 |
| --- |
| 29.41 |
| --- |
| 33.69 |
| --- |
| 31.87 |
| --- |
| 34.58 |
| --- |
| 34.58 |
| --- |
| 33.23 |
| --- |
| 32.69 |
| --- |
| 19.11 |
| --- |
| 17.54 |
| --- |
| 18.79 |
| --- |
| 14.08 |
| --- |
| 20.0 |
| --- |
| 14.83 |
| --- |
| 18.79 |
| --- |
| 14.58 |
| --- |
| 17.83 |
| --- |
| 17.9 |
| --- |
Figure S3: Effects of Lv-Drp1 on migration of 95D lung cancer cells.

## Slide 4
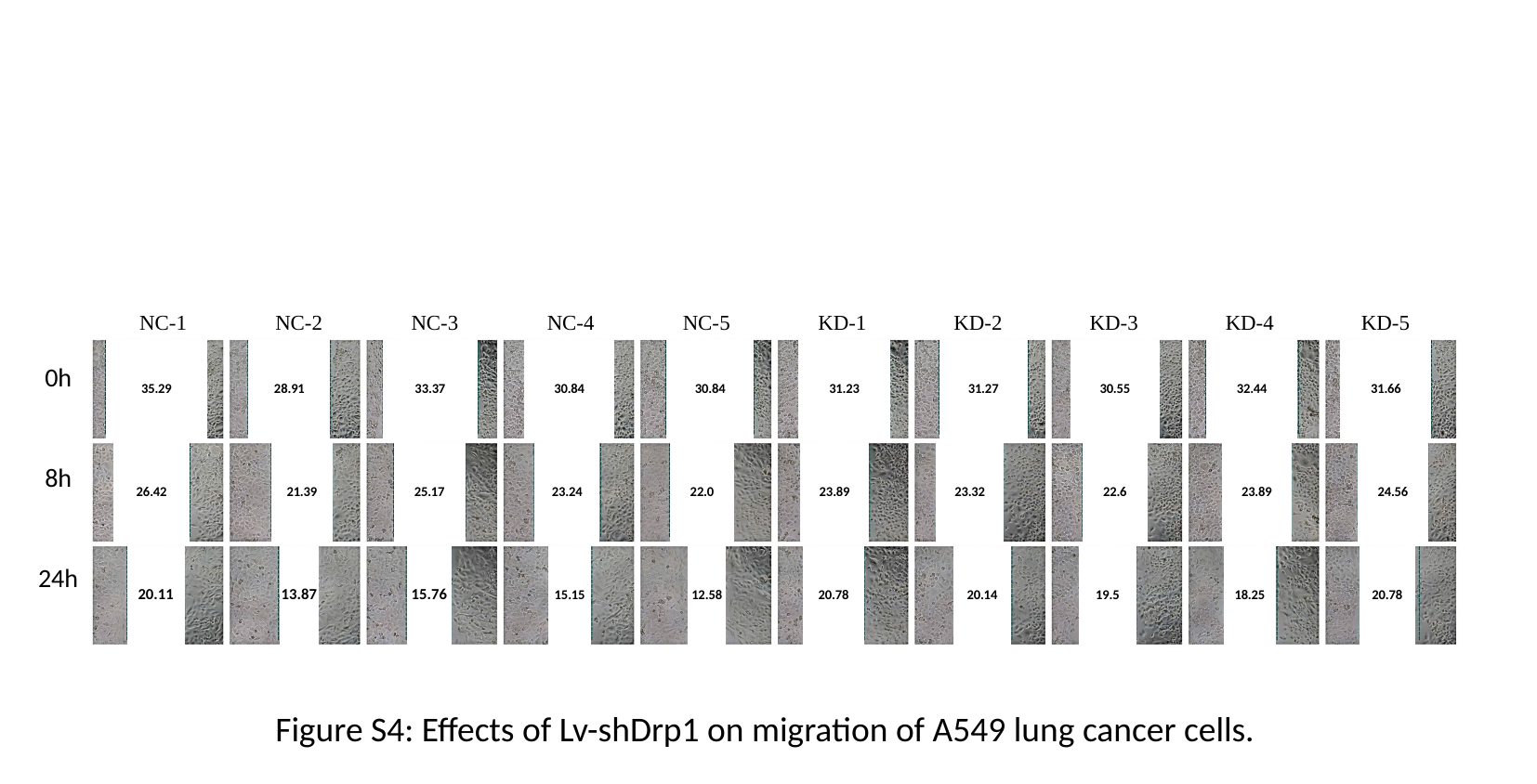

| NC-1 | NC-2 | NC-3 | NC-4 | NC-5 | KD-1 | KD-2 | KD-3 | KD-4 | KD-5 |
| --- | --- | --- | --- | --- | --- | --- | --- | --- | --- |
| 0h |
| --- |
| 8h |
| 24h |
| 35.29 |
| --- |
| 28.91 |
| --- |
| 33.37 |
| --- |
| 30.84 |
| --- |
| 30.84 |
| --- |
| 31.23 |
| --- |
| 31.27 |
| --- |
| 30.55 |
| --- |
| 32.44 |
| --- |
| 31.66 |
| --- |
| 26.42 |
| --- |
| 21.39 |
| --- |
| 25.17 |
| --- |
| 23.24 |
| --- |
| 22.0 |
| --- |
| 23.89 |
| --- |
| 23.32 |
| --- |
| 22.6 |
| --- |
| 23.89 |
| --- |
| 24.56 |
| --- |
| 20.11 |
| --- |
| 13.87 |
| --- |
| 15.76 |
| --- |
| 15.15 |
| --- |
| 12.58 |
| --- |
| 20.78 |
| --- |
| 20.14 |
| --- |
| 19.5 |
| --- |
| 18.25 |
| --- |
| 20.78 |
| --- |
Figure S4: Effects of Lv-shDrp1 on migration of A549 lung cancer cells.
